# Cross-Sectional and Longitudinal Performance of Non-Invasive Tests of Liver Fibrosis in Patients with Non-Alcoholic Fatty Liver Disease

**DOI:** 10.3390/jcm12020650

**Published:** 2023-01-13

**Authors:** Angelo Armandi, Chiara Rosso, Ramy Younes, Diana Julie Leeming, Morten A. Karsdal, Gian Paolo Caviglia, Nuria Pérez-Diaz-del-Campo, Daphne D’Amato, Amina Abdulle, Aurora Nicolosi, Gabriele Castelnuovo, Giorgio Maria Saracco, Davide Giuseppe Ribaldone, Elisabetta Bugianesi

**Affiliations:** 1 Department of Medical Sciences, University of Turin, 10126 Turin, Italy; 2 Nordic Bioscience A/S, Biomarkers & Research, 2730 Herlev, Denmark; 3 Division of Gastroenterology, Città Della Salute e Della Scienza University-Hospital, 10100 Turin, Italy

**Keywords:** ADAPT, liver fibrosis, NAFLD, PRO-C3, Liver stiffness, non-invasive scores, FIB-4, NFS

## Abstract

**Background and aims:** Non-invasive tests (NITs) are needed in clinical practice to replace histology for the identification of liver fibrosis and prognostication in Non-Alcoholic Fatty Liver Disease (NAFLD). Novel collagen-derived fibrogenesis markers including N-terminal type III collagen pro-peptide (PRO-C3) are among the most promising tools in this field. The aim of this study was to assess the diagnostic accuracy of PRO-C3, the derivative ADAPT score, and other NITs for the identification of advanced fibrosis (stages 3–4) and changes over 12 months of follow-up. **Methods:** In this longitudinal study, 96 patients with biopsy-proven NAFLD were evaluated at baseline, of which 50 underwent a follow-up visit after 12 months. Clinical-biochemical parameters, liver stiffness (LS) by transient elastography, PRO-C3, and other NITs (ADAPT, FIB-4, NFS, APRI) were collected at baseline and follow-up. **Results:** LS showed the best accuracy for the identification of advanced fibrosis, with Area under the Receiving Operator Curve (AUROC) 0.82 (0.73–0.89) for a cut-off value of 9.4 kPa. Among the other NITs, the ADAPT score showed the best accuracy, with AUROC 0.80 (0.71–0.88) for a cut-off of 5.02 (Se 62%, Sp 89%, PPV 74%, NPV 83%). The comparison between the AUROC of LS with that of ADAPT was not statistically different (DeLong test *p* value 0.348). At follow-up, LS was slightly reduced, whilst PRO-C3 displayed a significant increase from baseline median 11.2 ng/mL to 13.9 ng/mL at follow-up (*p* = 0.017). Accordingly, ADAPT score increased from median 5.3 to 6.1 (*p* = 0.019). The other NITs did not significantly change over 12 months. **Conclusions:** The ADAPT score shows the best performance among non-invasive scores for the identification of advanced fibrosis, not different from LS. Collagen-derived biomarker PRO-C3 and the derivative score ADAPT display significant changes over time, and may be useful tools for monitoring the progression of liver disease or assessing responses to treatments.

## 1. Introduction

Non-Alcoholic Fatty Liver Disease (NAFLD) is the most common chronic liver disease, affecting about 25% of the adult population and is tightly linked to the components of metabolic syndrome [1]. Only a subgroup of NAFLD patients bear significant intrahepatic necroinflammation (Non-Alcoholic Steatohepatitis, NASH) [2] with potential progression to advanced liver disease and its complications. Liver fibrosis represents the most important prognostic factor, and the detection of advanced fibrosis is of utmost relevance in clinical practice [3,4].

To overcome the need for liver biopsy, which remains the gold standard for fibrosis staging, several non-invasive scores have been suggested [5]. In fact, liver biopsy is burdened by potential poor tolerability by the patients, along with side effects, which may be serious (including haemothorax), high costs, and limited accessibility. Among non-invasive scores, Fibrosis score-4 (FIB-4), Aspartate aminotransferase (AST) to platelet index (APRI), and NAFLD fibrosis score (NFS) use biochemical, anthropometric, and clinical variables for a first-line assessment of fibrosis stage, with higher accuracy for advanced fibrosis, but are affected by a high rate of indeterminate results. Among all non-invasive tests, liver stiffness (LS) by transient elastography is the most accurate tool to identify fibrosis stages, and is recommended in the hepatology setting for the selection of candidates for liver biopsy [6].

Recently, collagen-derived biomarkers of liver fibrogenesis have been introduced as promising direct biomarkers for the detection of liver fibrosis [7]. In particular, N-terminal type III collagen pro-peptide (PRO-C3) has provided good accuracy for the detection of significant fibrosis [8,9], with the advantage of a direct assessment of the ongoing intrahepatic disease activity using a blood sample. In fact, collagen type III is upregulated in the early phases of liver fibrosis development, and PRO-C3 epitope levels in the blood are proportional to the amount of liver fibrogenesis, in particular with regards to advanced stages of fibrosis [10]. In addition, the recent score ADAPT, incorporating PRO-C3, has been developed for the detection of advanced fibrosis, showing the best performance among non-invasive scores [11].

We conducted a prospective study of biopsy-proven NAFLD patients to assess the diagnostic accuracy of non-invasive tests including LS, PRO-C3, and non-invasive scores (FIB-4, NFS, APRI, ADAPT) for the detection of advanced fibrosis, and their changes after a 12-month follow-up of observation.

## 2. Materials and Methods

### 2.1. Design of the Study and Characteristics of the Population

**Figure 1:**
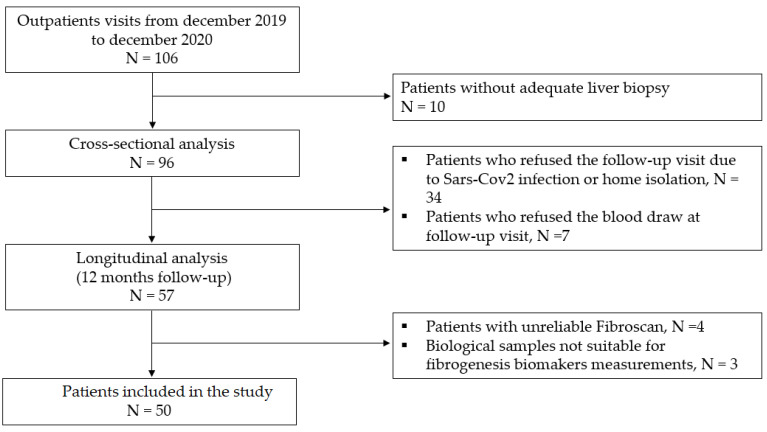
Flow-chart of the study.

A flow chart of the study is displayed in Figure 1.

This is a cohort study of 106 outpatients that consecutively underwent hepatologist consultation at the Liver Outpatient Clinic of the “Città della Salute e della Scienza di Torino” University Hospital, from December 2019 to December 2020. Inclusion criteria were age ≥18 years, and presence of NAFLD. NAFLD diagnosis was based on historical liver biopsy (performed within the previous 24 months), showing the typical histological features of liver injury (steatosis more than 5%, with or without hepatocellular ballooning and lobular inflammation). NASH was defined by the joint presence of steatosis, ballooning, and lobular inflammation, and was graded by the Non-Alcoholic Fatty Liver Disease Activity Score (NAS) [12]. Liver fibrosis was staged by Metavir score into four stages (F0–F4). Advanced fibrosis was defined by fibrosis stage >2. Exclusion criteria were significant alcohol intake (>30 gr/day for men and >20 gr/day for women) and concomitant presence of other etiologies of liver disease (including viral hepatitis, cholestatic or autoimmune hepatitis, iron or copper overload, and hepatotoxic medications).

A total of 96 patients were included in the cross-sectional analysis and were scheduled a follow-up visit. A follow-up visit was performed after 12 months from baseline. Of the 96 patients that were enrolled, 50 underwent a second hepatologist consultation and were included in the longitudinal analysis. Of the remaining patients, 34 refused to undergo an onsite visit during the SARS-CoV-2 pandemic outbreak; the other causes for missing follow-up are shown in Figure 1. NAFLD treatment consisted of standard recommendations for lifestyle changes, including both increased physical activity and dietary changes. No patients underwent bariatric surgery for the whole period of observation, nor were they enrolled in clinical trials with investigational products.

The study was carried out according to the principles of the Declaration of Helsinki, and it was approved by the Ethics Committee of the University Hospital “Città della Salute e della Scienza” of Torino (CEI/522, 23 December 2009). All patients signed an informed consent agreement for the participation in the study. All the subjects consented to having their personal data collected in a database, and to storage and use of their blood samples for research purposes.

### 2.2. Clinical and Laboratory Analysis

Clinical and biochemical parameters were collected at the time of the baseline and follow-up visit. LS was assessed within 7 days from the baseline and follow-up visit through transient elastography (Fibroscan. Echonsens, Paris, France). All measurements were conducted by an expert operator (G.P.C.) in fasting condition. A minimum of 10 measurements were taken for each patient, and IQR/med values <30% were considered technically reliable. M and XL probes were used when appropriate.

Plasma samples for laboratory investigations were collected at the time of baseline and follow-up visit, and were stored at −80 °C for all the analytical determinations. PRO-C3 was assessed by a competitive ELISA assay (Nordic Bioscience Laboratory, Herlev, Denmark) [13,14].

### 2.3. Non-Invasive Scores of Liver Fibrosis

The following scores were calculated for each patient according to the original formula: FIB-4 score [15], NFS [16], APRI [17]. In addition, we calculated the newly suggested score ADAPT, which includes PRO-C3 according to the following formula: exp[log_10_((age × PRO-C3)/rad(platelet))] + type 2 diabetes mellitus (T2DM) [11].

### 2.4. Statistical Analysis

Data are reported as mean and standard deviation (SD) for continuous normally distributed variables, as median and 95% confidence interval (CI) for the median for continuous not-normally distributed variables, or as frequency and percentage (%) for categorical variables. Comparisons between groups were performed by the Mann–Whitney test or by the Kruskal–Wallis test for non-normally distributed variables; a t-test was used for normally distributed variables. The Fisher’s exact test or the Chi-square test were used for categorical data. Spearman or Pearson correlations were performed as appropriate to evaluate the correlation between all the metabolic parameters. The Wilcoxon non-parametric test or the paired sample t-test for normally distributed variables were used as appropriate. Z-score was calculated to confirm real changes of clinical and biochemical variables over time. The diagnostic accuracy of the non-invasive test for the identification of advanced fibrosis (F3-4) was assessed by the area under the receiver operating curve (AUROC) analysis. Sensitivity (Se), specificity (Sp), positive predictive value (PPV), negative predictive value (NPV), positive likelihood ratio (LR+), and negative likelihood ratio (LR-) were reported for all parameters on the basis of the specific cut-off identified by the Youden index. The comparison between AUROCs was performed using the DeLong test. Values of *p* < 0.05 were considered statistically significant. All the analyses were performed with MedCalc Software bvba version 18.9.1 (Mariakerke, Belgium).

## 3. Results

### 3.1. Cross-Sectional Analysis

The baseline characteristics of the study cohort included in the cross-sectional analysis (*n* = 96) are shown in Table 1.

The median age was 49.5 (20.0–74.0) years and 62.2% were male. The median BMI was 28.4 (17.8–45.7) kg/m^2^, while arterial hypertension and T2DM were present in 25.5% and 30.6% of the total participants. The median LS was 7.9 (2.9–63.9) kPa. At histology, NASH was present in 74% of cases, while 32.6% had advanced fibrosis.

Overall, median FIB-4 score was 0.97 (0.26–4.74), median NFS was −2.18 (−5.33–2.81), and median APRI was 0.39 (0.13–2.86). Median serum PRO-C3 was 9.6 (4.0–61.6) ng/mL. The derivative ADAPT score had a median value of 3.99 (1.97–9.63). The distribution of non-invasive tests according to the stages of liver fibrosis is reported in Table 2.

The values of FIB-4 score and NFS, as well as ADAPT and serum PRO-C3 levels, were differently distributed across the histological fibrosis stages. In particular, PRO-C3 and ADAPT displayed a marked increase in the advanced fibrosis, when compared to lower stages of fibrosis (F0–2). Moreover, a good correlation was found between both PRO-C3 and ADAPT with respect to the other non-invasive scores, in particular FIB-4 score and NFS (r > 0.45, *p* < 0.0001). In addition, LS displayed the same positive correlation with regard to PRO-C3 (r = 0.45, *p* < 0.0001) and ADAPT (r = 0.48, *p* < 0.0001) (Supplementary Table S1).

The diagnostic accuracy of the non-invasive tests for the identification of the histological advanced fibrosis is reported in Table 3.

LS showed the best accuracy, with AUROC 0.82 (0.73–0.89) for a cut-off value of 9.4 kPa. Among the other tests, ADAPT score showed the best accuracy, with AUROC 0.80 (0.71–0.88) for a cut-off of 5.02 (Se 62%, Sp 89%, PPV 74%, NPV 83%). The comparison between the AUROC of LS with that of ADAPT was not statistically different (DeLong test *p* value 0.348) (Supplementary Figure S1 and Supplementary Table S2).

When combining LS with the other tests, ADAPT and NFS reached the best accuracy (AUROC 0.85 and 0.88), and ADAPT reached the best NPV among all tests (93%).

Additionally, we evaluated the diagnostic accuracy of non-invasive tests for the identification of advanced fibrosis (F > 2) (Supplementary Table S3; Supplementary Figure S2). In this population, LS confirmed the best accuracy, with AUROC 0.76 (0.75—0.83) for a cut-off value of 10.1 kPa, along with the combination of LS + NFS (AUROC 0.76 (0.66—0.84).

### 3.2. Longitudinal Analysis

A total of 50 patients were included in the 12-month follow-up analysis. A comparison between baseline and follow-up evaluation of clinical-anthropometric parameters of this subgroup is shown in Table 4.

**Figure 2:**
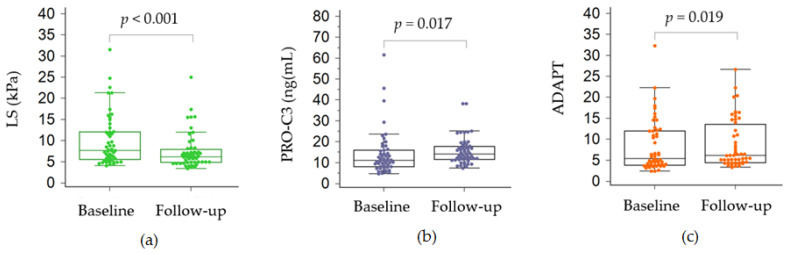
Comparison between baseline and follow-up values of liver stiffness (**a**), PRO-C3 (**b**) and ADAPT (**c**). ADAPT, PRO-C3 based score LS, liver stiffness; PRO-C3, N-terminal pro-peptide of type III collagen.

Overall, transaminases improved slightly, while LS showed a significant improvement, ranging from a median of 7.6 kPa to 6.2 kPa (*p* < 0.001). PRO-C3 values increased from median baseline 11.2 ng/mL to median 13.9 ng/mL at follow-up. Accordingly, ADAPT score increased from median 5.3 to 6.1 at follow-up (*p* = 0.019) (Figure 2).

In particular, LS showed a significant decrease in patients with advanced fibrosis (*p* = 0.018), while for patients in the F0–2 group, only a trend towards decrease was observed (*p* = 0.076). With regards to PRO-C3 and ADAPT, the increase from baseline to follow-up was observed only in the group of patients with F0–2 fibrosis (*p* = 0.0005 and *p* = 0.030, respectively). On the contrary, FIB-4 score, APRI and NFS did not show a difference from baseline to follow-up evaluation (Table 5).

We then divided the cohort into two subgroups, according to the follow-up changes in LS, and the difference between follow-up and baseline value was calculated (delta) for each non-invasive test. Overall, no significant changes were found between baseline and follow-up values for all parameters in both groups (Supplementary Table S4).

Additionally, we evaluated changes over time of non-invasive tests with regard to significant fibrosis (F > 2) (Supplementary Table S5). In particular, PRO-C3 and the ADAPT score showed a statistically significant difference (*p* = 0.005 and *p* = 0.006) in the F0-F1 group. Conversely, no changes were observed in the F2-F4 group. On the contrary, LS did not change in the F0-F1 group, but displayed a significant difference in the F2-F4 group (*p* = 0.0001).

## 4. Discussion

In this study of biopsy-proven NAFLD patients, we explored the accuracy of non-invasive tests to detect advanced fibrosis, and evaluated their changes over time. We found that FIB-4 score and NFS, as well as fibrogenesis marker PRO-C3 and the derivative ADAPT score, correlate with histological liver fibrosis, and show a stepwise increase across the fibrosis stages. LS remained the most accurate tool to predict advanced fibrosis, confirming the established evidence from the literature [5]. However, PRO-C3 alone showed comparable accuracy to the other scores (in particular FIB-4 score and NFS), and ADAPT resulted in the best accuracy among the scores, without statistical difference in comparison with LS. Interestingly, a combination of LS with ADAPT score provided the best negative predictive value to rule out advanced fibrosis.

The non-invasive scores of liver fibrosis are widely used in clinical practice as first-line tools including inexpensive, readily available parameters. The latest European guidelines on non-invasive tests recommend the use of FIB-4 score for the initial assessment of liver fibrosis, with the aim to refer properly to hepatologist evaluation [6]. However, these scores are burdened by about 30% of indeterminate values, and may not mirror the intrahepatic disease process. Collagen-derived markers of the interstitial or basement membrane may have the ability to identify the ongoing liver fibrogenesis, as part of the extracellular matrix turnover, which is enhanced in the inflammatory processes. In individuals with biopsy-based alcohol-related liver disease, the ADAPT score resulted as the best tool to predict advanced fibrosis, with an AUROC of 0.88 [18]. Similarly, in a large study conducted on two independent cohorts of biopsy-proven NAFLD patients, the ADAPT score reached the best accuracy for advanced fibrosis, with AUROC 0.86 in the derivation cohort and 0.87 in the validation cohort [11]. Moreover, post hoc data extrapolated from the CENTAUR phase II trial (Cenicriviroc for fibrosing NASH) further demonstrated that PRO-C3 had the ability to discriminate between simple fat accumulation and NASH, and was independently associated with liver fibrosis progression. In addition, the ADAPT score improved the ability of PRO-C3, with regard to both inflammation and fibrosis, and outperformed the other scores (FIB-4 scores, APRI, AST/ALT ratio) [19].

Similar to our findings, a population-based study showed a high negative predictive value (98%) to rule out advanced fibrosis [20], and the same results are observed when patients are selected according to the Metabolic-dysfunction Associated Fatty Liver Disease (MAFLD) definition [21,22]. In biopsied NAFLD patients, the sequential use of ADAPT and LS reached a diagnostic accuracy of 93%, supporting our results [20].

Interestingly, at the 12-month follow-up examination, we found that PRO-C3 and ADAPT displayed a significant change, increasing from baseline values, in particular in the group with mild or significant fibrosis. Overall, LS showed a reduction from baseline, but only in the group with advanced fibrosis. In addition, in the evaluation of the changes over time of each test within the group that either changed LS values or kept similar values, no difference was observed. LS gives a static picture of the severity of the liver injury, with regard to liver fibrosis. However, liver fibrogenesis is a highly dynamic process that is constantly modulated by the inflammatory processes in the presence of a harmful agent. The natural history of NAFLD is shaped by the intermittent action of multiple metabolic co-factors. In this cohort, no clear changes were detected with regard to metabolic dysfunctions after 12 months, as shown by the similarity in BMI values and in the lipid profile. Transaminases, as surrogates of liver inflammation, were not significantly altered, which may have provided the “static” follow-up values of the other scores where transaminases were included (FIB-4, APRI, NFS). The increases in PRO-C3 and ADAPT may have been the sole biochemical signs of the progressiveness of NAFLD. Moreover, the restriction of changes to mild-significant fibrosis stages may depend on the more florid inflammatory activity occurring in those stages.

The strength of this study is the high characterization of the cohort, and the biopsy-based diagnosis of NAFLD. However, the limited number of patients may have affected the relevance of the findings, in particular for follow-up evaluation. In addition, we selected consecutive patients from a pre-defined time interval. The historical biopsies of those patients were performed within the previous 24 months, which may have burdened the comparison with non-invasive tests and the evaluation of their diagnostic performance. In addition, lack of paired biopsies, as well as the dropout of patients at 12-month follow-up, limit the generalizability of our findings. Another limitation of this study is the absence of a comprehensive evaluation of all externally validated scores. In particular, we did not include Hepamet Fibrosis Score (HFS), which has proved the best accuracy in the identification of advanced fibrosis, additionally displaying a positive correlation with cardiovascular risk scores [23,24]. This aspect has a great relevance in clinical practice, potentially providing a simultaneous stratification of both advanced fibrosis and cardio-metabolic events, which represent the greatest burden in the NAFLD population.

In conclusion, the diagnostic accuracy of the ADAPT score is superior to the other non-invasive scores, and similar to LS, for the detection of advanced liver fibrosis in NAFLD patients. PRO-C3 and ADAPT score may represent valid tools for follow-up monitoring of liver disease severity, without the need for the instrumental support of hospital LS scanning.

## Figures and Tables

**Table 1:** Clinical, biochemical, and histological characteristics of the study cohort.

Variables	*n* = 96
Age (years), median (95% CI)	49.5 (20–74)
BMI (kg/m2), median (95% CI)	28.4 (17.8–45.7)
Male gender, *n* (%)	61 (62.2%)
Arterial hypertension, *n* (%)	23 (25.5%)
Type 2 diabetes mellitus, *n* (%)	30 (30.6%)
AST (IU/L), median (95% CI)	33 (12–198)
ALT (IU/L), median (95% CI)	53 (13–323)
Platelets (×10^9^/L), median (95% CI)	218 (101–474)
Albumin (g/dL), median (95% CI)	4.5 (4.2–4.6)
Triglycerides (mg/dL), median (95% CI)	121 (28–812)
Total cholesterol (mg/dL), median (95% CI)	187 (85–300)
HDL cholesterol (mg/dL), median (95% CI)	45 (16–75)
Liver stiffness, kPa	7.9 (3.9–63.9)
**Histological features**	
Steatosis grade, *n* (%) 1 2 3	49 (51%) 33 (34.4%) 14 (14.6%)
Hepatocellular ballooning, *n* (%) 0 1 2	10 (10.4%) 53 (55.2%) 33 (34.4%)
Lobular inflammation, *n* (%) 0 1 2	20 (20.8%) 65 (67.7%) 11 (11.5%)
Hepatic fibrosis F0/F1 F2 F3/F4	50 (51.1%) 16 (16.3%) 32 (32.6%)
NASH, *n* (%)	71 (74%)

ALT, alanine aminotransferase; AST, aspartate aminotransferase; BMI, body mass index; HDL, high density lipoprotein cholesterol; NASH, non-alcoholic steatohepatitis.

**Table 2:** Non-invasive tests according to the stages of hepatic fibrosis.

Non-Invasive Tests	F0/F1	F2	F3/F4	*p*-Value
FIB-4	0.86 (0.74–0.99)	0.92 (0.60–1.14)	1.22 (0.97–1.51)	0.0060
NFS	−2.67 (−3.17–−2.23)	−2.46 (−3.16–−1.89)	−0.88 (−1.32–−0.50)	<0.0001
APRI	0.36 (0.30–0.39)	0.39 (0.30–0.47)	0.44 (0.34–0.65)	0.0880
PRO-C3 (ng/mL)	8.3 (6.8–10.2)	8.6 (6.2–12.0)	12.6 (9.6–14.4)	0.0032
ADAPT	3.71 (3.23–4.05)	3.60 (3.34–3.99)	5.15 (4.51–5.73)	<0.0001

Data are reported as median and 95% confidence interval (CI) for the median. *p*-value refers to the difference between the three groups of fibrosis (F0/F1 vs. F2 vs. F3/F4). ADAPT, PRO-C3 based score; APRI, aspartate aminotransferase (AST) to platelet ratio index; FIB-4, fibrosis score 4; NFS, non-alcoholic fatty liver disease fibrosis score; PRO-C3, N-terminal propeptide of type III collagen.

**Table 3:** Diagnostic accuracy of FIB-4, NFS, APRI, PRO-C3, and ADAPT, alone or in combination with liver stiffness for the identification of advanced fibrosis.

Non-Invasive Tests	AUROC (95% CI)	Cut-Off	Se (%)	Sp (%)	PPV (%)	NPV (%)	LR+	LR−
FIB-4	0.721 (0.622–0.807)	1.27	53	85	63	79	3.51	0.55
NFS	0.799 (0.704–0.873)	−1.67	77	80	65	88	3.87	0.28
APRI	0.651 (0.548–0.745)	0.65	37	92	71	75	4.95	0.68
PRO-C3 (ng/mL)	0.732 (0.633–0.816)	11.3	62	76	56	81	2.58	0.50
ADAPT	0.804 (0.712–0.878)	5.02	62	89	74	83	5.89	0.42
LS, (kPa)	0.823 (0.733–0.893)	9.4	72	86	72	86	5.27	0.33
**Combinations**								
LS + FIB-4	0.831 (0.742–0.899)	0.32	69	89	76	85	6.48	0.35
LS + NFS	0.881 (0.799–0.938)	0.29	81	88	76	91	6.55	0.22
LS + APRI	0.824 (0.734–0.893)	0.32	69	89	76	86	6.48	0.35
LS + PRO-C3 (ng/mL)	0.823 (0.733–0.893)	0.25	75	77	61	86	3.30	0.32
LS + ADAPT	0.859 (0.775–0.921)	0.24	87	76	64	93	3.61	0.17

Cut-off values derived from the Youden index. ADAPT, PRO-C3 based score; APRI, aspartate aminotransferase (AST) to platelet ratio index; AUROC, area under the receiver operating characteristic curve; FIB-4, fibrosis score 4; LR+, positive likelihood ratio; LR−, negative likelihood ratio; LS, liver stiffness; NFS, non-alcoholic fatty liver disease fibrosis score; NPV, negative predictive value; PPV, positive predictive value; PRO-C3, N-terminal pro-peptide of type III collagen; Se, sensitivity; Sp, specificity.

**Table 4:** Comparison of clinical, anthropometric, and biochemical characteristics between baseline and follow-up.

Population (*n* = 50)	Baseline	Follow-Up	*z-*Score	*p*-Value
BMI, kg/m^2^	30 (28.3–31.3)	29.9 (29.0–30.8)	1.30	0.194
AST (IU/L), median (95% CI)	31 (27–36)	26 (23–30)	2.31	0.021
ALT (IU/L), median (95% CI)	46 (39–56)	34 (26–43)	3.49	<0.001
Platelets (×10^9^/L), median (95% CI)	223 (205–259)	234 (198–254)	1.07	0.284
Albumin (g/dL), median (95% CI)	4.5 (4.2–4.6)	4.6 (4.5–4.6)	−1.28	0.200
Triglycerides (mg/dL), median (95% CI)	130 (122–139)	122 (114–133)	1.34	0.179
Total-Chol (mg/dL), median (95% CI)	184 (175–195)	185 (178–188)	0.58	0.562
HDL-Chol (mg/dL), median (95% CI)	42 (40–50)	47 (45–48)	−2.69	0.007
LS, kPa	7.6 (6.4–9.6)	6.2 (5.2–6.9)	4.18	<0.001

ALT, alanine aminotransferase; AST, aspartate aminotransferase; BMI, body mass index; Chol, cholesterol; HDL, high density lipoprotein cholesterol; LS, liver stiffness.

**Table 5:** Comparison of non-invasive tests between baseline and follow-up.

	F0–F2 (*n* = 31)			F3–F4 (*n* = 19)		
Non-Invasive Scores	Baseline	Follow-Up	*p* Value	Baseline	Follow-Up	*p* Value
LS, kPa	6.3 (4.9–8.3)	5.7 (4.6–6.9)	0.0760	12 (8.4–16.2)	7.3 (6.1–11.5)	0.0180
FIB-4	0.76 (0.52–1.06)	0.83 (0.59–1.08)	0.7143	1.28 (0.97–2.32)	1.20 (1.01–1.76)	0.8609
NFS	0.69 (−0.27–1.37)	0.64 (−0.16–2.15)	0.6625	2.82 (1.42–3.51)	2.95 (1.82–3.49)	0.9534
APRI	0.30 (0.20–0.36)	0.28 (0.20–0.38)	0.9215	0.61 (0.34–0.90)	0.37 (0.29–0.47)	0.0681
PRO-C3, ng/mL	9.9 (6.5–13.0)	14.1 (11.3–16.7)	0.0005	14.4 (10.6–23.5)	13 (11.7–23.4)	0.8839
ADAPT	4.3 (3.6–5.8)	5.1 (4.4–6.3)	0.0301	11.3 (6.6–17)	12.1 (6.7–16.2)	0.9884

Data are reported as median and 95% confidence interval (CI). ADAPT, PRO-C3 based score; APRI, aspartate aminotransferase (AST) to platelet ratio index; FIB-4, fibrosis score 4; NFS, non-alcoholic fatty liver disease fibrosis score; PRO-C3, N-terminal propeptide of type III collagen.

## Data Availability

The data presented in this study are available on request from the corresponding author.

## Supplementary Materials

The following supporting information can be downloaded at: https://www.mdpi.com/article/10.3390/jcm12020650/s1, Figure S1: Comparison of the area under the receiver operating characteristic curve of non-invasive scores, PRO-C3 and liver stiffness for advanced fibrosis; Figure S2: Comparison of the area under the receiver operating characteristic curve of non-invasive scores, PRO-C3 and liver stiffness for significant fibrosis. Table S1: Correlations between PRO-C3 and ADAPT with the other non-invasive scores of hepatic fibrosis and Fibroscan; Table S2: Comparison between AUROCs; Table S3: Diagnostic accuracy of FIB-4, NFS, APRI, PRO-C3 and ADAPT alone or in combination with liver stiffness for the identification of significant fibrosis (F > 2). Table S4: Differences between baseline and follow-up variables according to the follow-up change in liver stiffness. Table S5: Comparison of non-invasive tests between baseline and follow-up, stratified for significant fibrosis.
