# Supplementary material for: Cross-Sectional and Longitudinal Performance of Non-Invasive Tests of Liver Fibrosis in Patients with Non-Alcoholic Fatty Liver Disease
Source: J Clin Med. 2023 Jan 13;12(2):650. doi: 10.3390/jcm12020650 (PMC9862059; doi:10.3390/jcm12020650)
Supplement: Supplementary file 1 [file jcm-12-00650-s001.zip › jcm-1998389-supplementary.pdf]

Supplementary Table S1. Correlations between PRO-C3 and ADAPT with the other non-invasive scores of hepatic fibrosis and Liver Stiffness.

Non-invasive scores	PRO-C3 (ng/mL)	ADAPT
	r_S<br>*p*-Value	r_S<br>*p*-Value
FIB-4	0.49<br><0.0001	0.65<br><0.0001
NFS	0.47<br><0.0001	0.81<br><0.0001
APRI	0.46<br><0.0001	0.10<br>0.3232
Liver stiffness (kPa)	0.45<br><0.0001	0.48<br><0.0001

Data are reported as Spearman coefficient (r_S) and *p*-Value. ADAPT, PRO-C3 based score; APRI, aspartate aminotransferase (AST) to platelet ratio index; FIB-4, fibrosis score 4; NFS, non-alcoholic fatty liver disease fibrosis score; PRO-C3, N-terminal propeptide of type III collagen.

Supplementary Table S2. Comparison between AUROCs.

Δ variables	Δ AUROCs (95% CI)	*p*-Value
LS *vs.* NFS	0.035 (-0.102-0.171)	0.6175
LS *vs.* PRO-C3	0.095 (-0.032-0.222)	0.1437
LS *vs.* ADAPT	0.020 (-0.106-0.145)	0.7562
**Combinations**		
LS *vs.* LS + NFS	0.051 (-0.031-0.134)	0.223
LS *vs.* LS + PRO-C3	0.004 (-0.029-0.037)	0.8147
LS *vs.* LS + ADAPT	0.033 (-0.047-0.114)	0.4202

Differences between the AUROCs were assessed by DeLong test. ADAPT, PRO-C3 based score; LS, liver stiffness; NFS, non-alcoholic fatty liver disease fibrosis score; PRO-C3, N-terminal pro-peptide of type III collagen.

Supplementary Table S3. Diagnostic accuracy of FIB-4, NFS, APRI, PRO-C3 and ADAPT alone or in combination with liver stiffness for the identification of significant fibrosis (F > 2).

Non-invasive tests	AUROC (95% CI)	Cut-off	Se (%)	Sp (%)	PPV (%)	NPV(%)	LR+	LR-
FIB-4	0.643 (0.539-0.738)	1.04	56	70	63	64	1.88	0.62
NFS	0.698 (0.594-0.788)	-2.05	64	71	67	69	2.26	0.50
APRI	0.619 (0.514-0.716)	0.39	56	64	59	61	1.57	0.68
PRO-C3 (ng/mL)	0.654 (0.550-0.749)	5.4	100	26	55	100	1.35	-
ADAPT	0.681 (0.578-0.772)	4.68	50	82	72	64	2.78	0.61
LS, (kPa)	0.757 (0.659-0.839)	10.1	46	94	88	65	7.61	0.58
**Combinations**								
LS + FIB-4	0.743 (0.644-0.827)	0.53	50	92	85	67	6.25	0.54
LS + NFS	0.764 (0.665-0.845)	0.46	60	84	77	70	3.68	0.48
LS + APRI	0.753 (0.655-0.835)	0.53	52	92	86	68	6.52	0.52
LS + PRO-C3 (ng/mL)	0.749 (0.650-0.832)	0.43	65	72	68	69	2.33	0.48
LS + ADAPT	0.747 (0.648-0.831)	0.59	46	96	91	66	11.4	0.67

Cut-off values derived from the Youden index. ADAPT, PRO-C3 based score; APRI, aspartate aminotransferase (AST) to platelet ratio index; AUROC, area under the receiver operating characteristic curve; FIB-4, fibrosis score 4; LR+, positive likelihood ratio; LR-, negative likelihood ratio; LS, liver stiffness; NFS, non-alcoholic fatty liver disease fibrosis score; NPV, negative predictive value; PPV, positive predictive value; PRO-C3, N-terminal pro-peptide of type III collagen; Se, sensitivity; Sp, specificity.

Supplementary Table S4. Differences between baseline and follow-up variables according to the follow-up change in liver stiffness.

	No change in LS	Improvement in LS	
Δ variables	Median (95% CI)	Median (95% CI)	*p*-Value
Δ FIB-4	-0.06 (-0.18-0.22)	0.05 (-0.09-0.25)	0.284
Δ NFS	-0.11 (-0.37-0.20)	-0.14 (-0.34-0.36)	0.856
Δ APRI	0.02 (-0.03-0.10)	0.06 (0.01-0.27)	0.275
Δ PRO-C3 (ng/mL)	-2.85 (-4.50- -0.95)	-1.45 (-6.00-2.20)	0.649
Δ ADAPT	-0.5 (-1.30- -0.10)	-0.25 (-1.50-0.90)	0.716

ADAPT, PRO-C3 based score; ALT, alanine aminotransferase; AST, aspartate aminotransferase; BMI, body mass index; Chol, cholesterol; FIB-4, fibrosis score 4; HDL, high density lipoprotein cholesterol; NFS, non-alcoholic fatty liver disease fibrosis score; PRO-C3, N-terminal propeptide of type III collagen.

Supplementary Table S5. Comparison of non-invasive tests between baseline and follow-up, stratified for significant fibrosis.

	**F0–F1 (N = 22)**			**F2–F4 (N = 28)**		
**Non-invasive scores**	**Baseline**	**Follow up**	***P value***	**Baseline**	**Follow up**	***P value***
LS, kPa	5.8 (4.9-7.7)	5.5 (4.6-7.0)	0.1327	11.2 (7.4-15.2)	6.2 (5.0-9.1)	0.0001
FIB-4	0.75 (0.52-0.99)	0.79 (0.58-1.02)	0.5057	1.11 (0.76-1.58)	1.15 (0.87-1.74)	0.6488
NFS	0.76 (-0.30- 1.38)	0.74 (-0.02-2.05)	0.6148	2.00 (0.75-3.36)	2.24 (0.87-3.36)	0.3016
APRI	0.28 (0.19-0.35)	0.28 (0.19-0.38)	0.2113	0.41 (0.29-0.71)	0.33 (0.29-0.47)	0.1791
PRO-C3, ng/mL	9.9 (6.3-13.2)	13.3 (11.2-16.3)	0.005	12.5 (9.1-18.6)	14.2 (11.9-19.4)	0.4123
ADAPT	4.5 (3.5-5.9)	5.1 (4.3-6.2)	0.006	9.9 (4.1-13.7)	8.2 (5.2-15.3)	0.3550

Data are reported as median and 95% confidence interval (CI). ADAPT, PRO-C3 based score; APRI, aspartate aminotransferase (AST) to platelet ratio index; FIB-4, fibrosis score 4; NFS, non-alcoholic fatty liver disease fibrosis score; PRO-C3, N-terminal propeptide of type III collagen.

Supplementary Figure S1. Comparison of the area under the receiver operating characteristic curve of non-invasive scores, PRO-C3 and liver stiffness for advanced fibrosis.

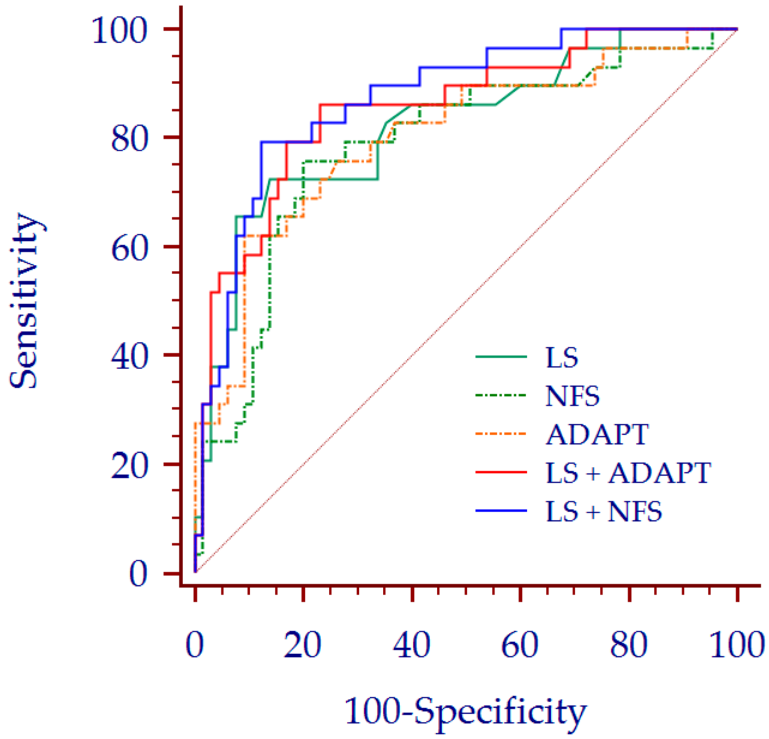

ADAPT, PRO-C3 based score; LS, liver stiffness; NFS, non-alcoholic fatty liver disease fibrosis score.

Supplementary Figure S2. Comparison of the area under the receiver operating characteristic curve of non-invasive scores, PRO-C3 and liver stiffness for significant fibrosis.

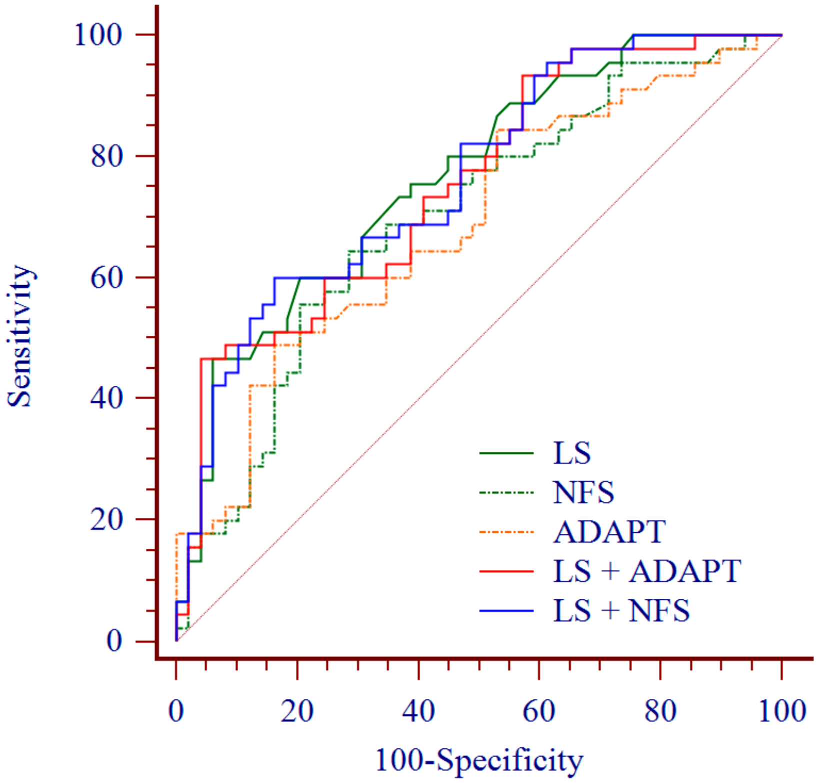

ADAPT, PRO-C3 based score; LS, liver stiffness; NFS, non-alcoholic fatty liver disease fibrosis score.
